# Association between Single Nucleotide Polymorphisms in the *ADD3* Gene and Susceptibility to Biliary Atresia

**DOI:** 10.1371/journal.pone.0107977

**Published:** 2014-10-06

**Authors:** Shuaidan Zeng, Peng Sun, Zimin Chen, Jianxiong Mao, Jianyao Wang, Bin Wang, Lei Liu

**Affiliations:** 1 Zhuhai Campus of Zunyi Medical College, Zhuhai, Guangdong, China; 2 Department of General Surgery, Shenzhen Children's Hospital, Shenzhen, Guangdong, China; MOE Key Laboratory of Environment and Health, School of Public Health, Tongji Medical College, Huazhong University of Science and Technology, China

## Abstract

**Background and Objectives:**

Based on the results of previous studies, the *ADD3* gene, located in the 10q24.2 region, may be a susceptibility gene of biliary atresia (BA). In this study, two single nucleotide polymorphisms (SNPs) in the *ADD3* gene, rs17095355 C/T and rs10509906 G/C, were selected to investigate whether there is an association between these SNPs and susceptibility to BA in a Chinese population.

**Methods:**

A total of 752 Han Chinese (134 BA cases and 618 ethnically matched healthy controls) were included in the present study. The *ADD3* gene polymorphisms were genotyped using a TaqMan genotyping assay.

**Results:**

Positive associations were found for the SNP rs17095355 in the codominant model; specifically, the frequencies of the CT and TT genotypes and the T allele were higher in the cases than the controls, demonstrating a significant risk for BA (odds ratio [OR] = 1.62, 95% confidence interval [CI] = 1.02–2.58; OR = 2.89, 95% CI = 1.72–4.86; and OR = 1.75, 95% CI = 1.34–2.29, respectively). Regarding rs10509906, the per-C-allele conferred an OR of 0.70 (95% CI = 0.49–1.00) under the additive model. A greater risk of BA was associated with the T_a_-G_b_ (a for rs17095355 and b for rs10509906) haplotype (OR = 1.82, 95% CI = 1.27–2.61) compared with the C_a_-C_b_ haplotype.

**Conclusion:**

This study suggests that the *ADD3* gene plays an important role in BA pathogenesis and reveals a significant association between two SNPs, rs17095355 and rs10509906, and BA.

## Introduction

Biliary atresia (BA) is a devastating disease of infancy that invariably leads, if left untreated, to cirrhosis, liver failure, and death. Similar to North America and Western Europe, the incidence in the UK is approximately 1 in 16,000 neonates; the incidence is much higher in parts of Asia, including Japan and, most likely, China, estimated at approximately 1 in 10,000 neonates. Conjugated jaundice, pale acholic stools, and dark urine are the primary clinical manifestations of BA [1]. The initial treatment for BA, Kasai radical surgery, needs to be performed within the first 3 months of life to achieve a better outcome. If the surgery fails to reconstruct a new biliary system, the patient will die at approximately 1 year of age due to liver failure and other serious complications. Liver transplantation is a potential solution, but only a few patients can receive a new liver because of the insufficient liver source in China. The etiology of BA is still unknown, but several hypotheses have been considered, including perinatal virus infection, congenital and acquired immune injury, maternal microchimerisms in the liver that cause a graft-vs-host reaction, the inherited pathogenic factor hypothesis, and ductal plate malformation [2]–[8]. Moreover, genetic factors are strongly suggested to play an important role in BA. In the past 10 years, researchers have identified a number of genes associated with BA, such as the migration inhibitory factor (*MIF*), *CD14*, intercellular adhesion molecule-1 (*ICAM-1*), adiponectin (*APM1*), and ITGB2 (*CD18*) [9]–[13].

In 2010, a Chinese population-based genome-wide association study (GWAS) was performed by Garcia-Barceló et al. [14]. Rs17095355, which maps to the intergenic region between *ADD3* (adducin 3) and *XPNPEP1* (X-prolyl aminopeptidase 1), was identified in a GWAS of 200 patients and 481 controls. This finding was supported by the replication genotyping of the 10 most BA-associated SNPs in 124 cases and 90 controls, with the strongest overall association found in 10q24.2 (*P* = 6.94×10^−9^). This SNP was subsequently replicated in the Thai population (*P*<0.002) [15].

However, Cheng et al. [16] accomplished a fine-mapping study of the BA-associated region in 2013 in a Han Chinese population. This study revealed a common risk haplotype composed of 5 tag SNPs, including rs17095355, rs10509906, rs2501577, rs6584970, and rs7086057; rs10509906 on the common protective haplotype CCATA was also independently associated with BA. Moreover, they also found that the BA-associated potentially regulatory SNPs correlated with *ADD3* gene expression in bioinformatics and *in vivo* genotype-expression investigations.

With the aim of investigating the association between the *ADD3* gene polymorphisms and susceptibility to BA, we conducted a case-control study to verify the effects of rs17095355 and rs10509906 in an independent Chinese sample.

## Materials and Methods

### 1. Study Population

From 2010 to 2013, 134 unrelated children were diagnosed with BA by laparoscopic cholangiography and a biopsy of the liver and the extrahepatic biliary tree at the Shenzhen Children's Hospital. Parental written consent was given. This study included 66 males and 68 females with an average age of 72 days (range 46–123) at the time of surgery. None of the patients had any other associated congenital malformations. All patients underwent the Roux-en-Y hepaticojejunostomy reconstruction successfully, and no serious postoperative complications were noted during hospitalization.

For the controls, 618 individuals (303 males and 315 females) of southern Chinese origins without a diagnosis of BA, congenital disease, or liver disease were included. Written informed consent to take blood samples from the children was obtained from all the subjects and the legal guardians of every child. The study protocol conformed to the ethical guidelines of the 1975 Declaration of Helsinki and was approved by the Ethics Committee on Human Research of the Faculty of the Shenzhen Children's Hospital.

### 2. Genotyping

The genomic DNA was extracted from peripheral blood leukocytes or liver tissue collected during the surgery of the BA children and from peripheral blood leukocytes of the healthy controls using a DNeasy Blood & Tissue Kit (Qiagen, Hilden, Germany). The genomic DNA samples were stored at −20°C for further analysis. The genotypes of rs17095355 and rs10509906 were determined using a TaqMan SNP Genotyping Assay (Applied Biosystems, Foster City, CA, USA). Polymerase chain reaction (PCR) was carried out in a 384-well GeneAmp PCR System 9700 (Applied Biosystems) with mixtures consisting of 1 µl of DNA (10 ng/µl), 2.5 µl of TaqMan genotyping master mix, 0.125 µl of TaqMan MGB probes (containing distinct fluorescent dyes and a PCR primer pair), and ddH_2_O, with a final volume of 5 µl. The thermal cycling conditions were as follows: denaturation at 95°C for 10 min, followed by 50 cycles of denaturation at 92°C for 15 s and annealing and extension at 60°C for 90 s. After PCR, the TaqMan assay plates were transferred to the ABI PRISM 7900 Sequence Detection System (Applied Biosystems), where the endpoint fluorescence intensity in each well of the plate was read. The allelic-specific fluorescence data from each plate were analyzed using the SDS v2.4 software (Applied Biosystems) to automatically determine each genotype.

### 3. Statistical Analysis

The χ^2^ test was performed to estimate the differences in the variables and the distributions of the genotypes between the cases and controls. Hardy-Weinberg equilibrium was evaluated using the goodness-of-fit χ^2^ test in the controls, and a value of *P*<0.05 was considered to indicate significant disequilibrium. The association between the case-control status and each SNP was assessed by the odds ratio (OR) and the corresponding 95% confidence interval (CI). To avoid the assumption of genetic models, codominant, dominant, recessive, and additive models were all analyzed. A stepwise procedure was performed to control the false discovery rate (FDR), which was applied for multiple comparison correction. The linkage disequilibrium (LD) of the candidate SNPs and the haplotype frequencies were estimated using HaploView V4.2 and PHASE V2.0 software, respectively. The *r^2^* value was used to measure the degree of linkage disequilibrium. The ORs and 95% CIs, adjusted by gender, were calculated with an unconditional logistic regression. The statistical analyses were performed using SPSS software V.20.0 (SPSS, Chicago, Illinois, USA); a *P*<0.05 was considered statistically significant.

## Results

### 1. Population characteristics

A total of 134 incident cases of BA and 618 controls were enrolled in this study. The genotype distributions of rs17095355 and rs10509906 in the controls were in Hardy-Weinberg equilibrium (*P* = 0.24 and *P* = 0.27). The male to female ratios of the cases and controls were 0.97 (66/68) and 0.96 (303/315), respectively, and there was no significant difference in gender distribution between the patients and controls (*P* = 0.962). Genotyping was successful in a total of 133 cases (99%) for rs17095355, 129 cases (96%) for rs10509906, and all of the controls.

### 2. Association analysis

Regarding the two SNPs investigated, after correction for multiple comparisons by FDR, rs17095355 showed a significant association with BA in all of the models, while rs10509906 exhibited no significant association with BA under every model. The detailed genotype frequencies of rs17095355 among the 618 controls and 133 cases are shown in Table 1. In the unconditional logistic regression analysis, the individuals with CT and TT genotypes had a significantly increased risk of BA (*P_FDR_* = 0.043, OR = 1.62, 95% CI = 1.02–2.58; *P_FDR_* = 1.95×10^−4^, OR = 2.89, 95% CI = 1.72–4.86, respectively) compared with those with the CC homozygote genotype. The dominant and recessive models were analyzed, and the genotypic models (CT plus TT vs CC) and (TT vs CC plus CT) showed a significant association with BA (*P_FDR_* = 2.40×10^−3^, OR = 1.97, 95% CI = 1.27–3.03; *P_FDR_* = 5.73×10^−4^, OR = 2.15, 95% CI = 1.41–3.29, respectively). The C and T allele frequencies of the controls were 60.1% and 39.9%, respectively, and the T allele was also found to be associated with an increased risk for BA (*P_FDR_* = 2.22×10^−4^, OR = 1.75, 95% CI = 1.34–2.29). Similarly, a positive result was found in the additive model, with a per-T-allele OR of 1.70 (95% CI = 1.31–2.20, *P_FDR_* = 1.44×10^−3^).

**Table 1:** Associations between rs17095355 and BA risk in a Chinese population.

	Controls (%)	Cases (%)	*P* #	OR (95% CI)	*P* +	*P_FDR_* *
**rs17095355**	618	133				
CC	231(37.4)	31(23.3)		1.00(reference)		
CT	281(45.5)	61(45.9)		1.62(1.02–2.58)	0.043	0.043
TT	106(17.2)	41(30.8)	<0.001	2.89(1.72–4.86)	6.50×10^−5^	1.95×10^−4^
Dominant model	387(62.6)	102(76.7)	0.002	1.97(1.27–3.03)	2.00×10^−3^	2.40×10^−3^
Recessive model	512(82.8)	92(69.2)	<0.001	2.15(1.41–3.29)	3.82×10^−4^	5.73×10^−4^
Allele C	743(60.1)	123(46.2)		1.00(reference)		
Allele T	493(39.9)	143(53.8)	<0.001	1.75(1.34–2.29)	3.70×10^−5^	2.22×10^−4^
Additive model				1.70(1.31–2.21)	7.20×10^−5^	1.44×10^−3^

# *P* values were computed by the Pearson chi-square test.

+ Data were calculated by logistic regression after adjusting for gender.

*P values were modified by the FDR correction for multiple comparisons.

When comparing the cases with controls, we observed no statistically significant differences in genotype (*P* = 0.062) or allele (*P* = 0.053) distributions of rs10509906 (Table 2). Additionally, the C allele seems to reduce the risk for BA (*P_FDR_* = 0.108, OR = 0.72, 95% CI = 0.51–1.00). An independent effect of rs10509906 was formally test by the logistic regression analysis of the two SNPs to verify significance of the rs10509906 after inclusion of the rs17095355, however, no significant difference was found in each model.

**Table 2:** Associations between rs10509906 and BA risk in a Chinese population.

	Controls (%)	Cases (%)	*P* #	OR (95% CI)	*P* +	*P_FDR_* *	OR_b_ (95% CI)	*P_b_* +	*P_FDR_* *
**rs10509906**	618	129							
GG	350(56.6)	82(63.6)		1.00(reference)			1.00(reference)		
CG	237(38.3)	46(35.7)		0.83(0.56–1.23)	0.353	0.353	1.15(0.74–1.79)	0.541	0.901
CC	31(5.0)	1(0.8)	0.062	0.14(0.02–1.02)	0.053	0.159	0.23(0.03–1.75)	0.155	0.388
Dominant model	268(43.4)	47(36.4)	0.147	0.75(0.51–1.11)	0.148	0.178	0.91(0.60–1.38)	0.649	0.811
Recessive model	587(95.0)	128(99.2)	0.030	6.84(0.92–50.57)	0.060	0.090	5.67(0.77–42.23)	0.089	0.445
Allele G	937(75.8)	210(81.4)		1.00(reference)					
Allele C	299(24.2)	48(18.6)	0.053	0.72(0.51–1.01)	0.054	0.108	0.97(0.66–1.42)	0.869	0.869
Additive model				0.70(0.49–1.00)	0.047	0.282			

# *P* values were computed by the Pearson chi-square test.

+ Data were calculated by logistic regression after adjusting for gender.

b: Logistic regression analysis of the independent effect of rs10509906 after inclusion of the rs17095355.

*P values were modified by the FDR correction for multiple comparisons.

### 3. Haplotypes and risk of BA

We did not observe LD between rs17095355 and rs10509906 (*r^2^* = 0.173). The *ADD3* haplotypes in the cases and controls were constructed and the results are shown in Table 3. The distribution of haplotype frequencies was significantly different between the cases and controls (*P*<0.001). Compared with the low-risk C_a_-C_b_ (a for rs17095355 and b for rs10509906) haplotype, the adjusted ORs for the T_a_-C_b_ and C_a_-G_b_ haplotypes were 0.65 (95% CI = 0.08–4.91) and 1.04 (95% CI = 0.71–1.54), respectively. The last haplotype containing the high-risk alleles T_a_ and G_b_ was associated with an increased risk for BA (OR = 1.82, 95% CI = 1.27–2.61) (*P*_trend_<0.001).

**Table 3:** Risk estimates for the extended *ADD3* haplotypes in the BA cases and controls.

	Controls (n = 618)	Cases (n = 134)			
Haplotype	No. of chromosomes (%)	No. of chromosomes (%)	*P* #	OR (95% CI)	*P* +
C_a_-C_b_	289(23.4)	47(17.5)		1.00(reference)	
T_a_-C_b_	10(0.8)	1(0.4)		0.62(0.08–4.91)	0.646
C_a_-G_b_	454(36.7)	77(28.7)		1.04(0.71–1.54)	0.831
T_a_-G_b_	483(39.1)	143(53.4)	<0.001	1.82(1.27–2.61)	<0.001
*P* for trend				<0.001	

a: rs17095355 b: rs10509906.

# *P* values were computed by the Pearson chi-square test.

+ Data were calculated by logistic regression after adjusting for gender.

## Discussion

In the current study, we investigated the association of rs17095355 with BA risk. Our results suggested that the rs17095355 SNP was significantly associated with an enhanced BA risk under the genotypic, dominant, recessive, and additive models, while the other SNP, rs10509906, only presented a significant protective effect under the additive model. In the haplotype analysis, we found that these two SNPs had a certain interaction within a haplotype to influence the risk of BA; specifically, the T_a_-G_b_ haplotype was associated with an increased risk of BA compared with the C_a_-C_b_ haplotype.

The etiology and pathogenesis of BA are currently unknown. While other hypotheses remain, with the development of genotyping technologies and the discovery of inherited pathogenic factors in BA, increasingly more researchers are focusing on the genes and SNPs that are associated with BA. The GWAS, as reported by Garcia-Barcelo et al., revealed a relationship between rs17095355, located in 10q24, and BA; this conclusion was a milestone in this field. Because rs17095355 has been shown to fall within the intergenic region of the *ADD3* and *XPNPEP1* genes, Garcia-Barceló et al. further tried to determine whether the SNP most associated with BA regulated the *ADD3* or *XPNPEP1* genes. The results revealed that the C>T transition at rs17095355 did not appear to have a functional effect, and no evidence linked the other BA-associated SNPs with the regulation of *ADD3* or *XPNPEP1* [14].

Cheng, et al. conducted a fine-mapping study that revealed a common haplotype in 10q24.2 that was associated with BA risk. Rs10509906 had a significant effect (*P* = 7.97×10^−4^) and was detected in the common protective haplotype CCATA. This study revealed that the risk alleles were associated with a reduced *ADD3* expression level in BA livers and that the genotype-*ADD3*-expression correlation only existed in BA livers, not in the non-BA livers. *ADD3* was also found to be expressed in biliary epithelia. Therefore, *ADD3* may play a role in managing biliary epithelia and its deregulation; this most likely result in a congenital bile duct defect, which can biologically influence BA. Additionally, they reported that the risk haplotype was correlated with *ADD3* but not with *XPNPEP1* [16]. In addition, a replication of the GWAS was conducted in a Caucasian population by Tsai et al. [17], and the expression data in this study suggested that only *ADD3* is differentially expressed in BA patients.

Due to the difference in expression that only exists for the *ADD3* gene, we considered *ADD3* to be the key gene in the development of BA. *ADD3* encodes adducin 3, which is a member of the membrane skeletal proteins that are involved in the assembly of the spectrin-actin network in erythrocytes and is found at the sites of cell-cell contact in epithelial tissues [18], including the liver and bile ducts; it is also more abundantly expressed in the fetal liver than in the adult liver. Contractions of the bile canalicular membrane are controlled by actin-myosin interactions, and damage to these interaction mechanisms causes cholestasis. Increased actin and myosin deposition around the bile canaliculi has been observed in BA patients with bile discharge mechanism dysfunction after surgery. Additionally, the smooth muscle actin expression intensity influences the degree of fibrosis in patients with BA [14], [19]–[21].

Our study verified the results of the GWAS regarding rs17095355. The C and T allele frequencies of the controls are similar to the Chinese data in HapMap (58.3% and 41.7%). In the logistic regression analysis, the CT and TT genotypes of the dominant model (CT plus TT) and the T allele of rs17095355 were all associated with a more significant risk for BA than the wild-type homozygous CC genotype. This result means that individuals carrying the risk allele of rs17095355 have a higher susceptibility to BA. In the additive model, we observed that each T allele of rs17095355 increased the OR value of the risk for BA. As for rs10509906, our minor C allele frequency in the controls was 24.2%, which was similar to MAF 16.7% from HapMap. The C allele, the heterogeneous mutation CG, and the homozygous mutation CC of rs10509906 were associated with a non-significantly reduced risk for BA (Table 2), possibly because of our small sample size. However, we found that an additional C allele of rs10509906 marginally decreased the risk of BA. Furthermore, the results of the logistic regression analysis of the two SNPs showed that, even the rs10509906 was adjusted by the rs17095355, it still presented a negative effect on BA risk, which means there is no independent effect of the rs10509906 in our study. Nevertheless, the tendency yielded by the additive model and the marginal effect, yielded by the recessive model imply that the polymorphism rs10509906 may play a protective role in BA, similar to the results of the fine-mapping study conducted by Cheng, et al.

To understand how the haplotypes rs17095355 and rs10509906 contribute to the risk of BA, we conducted a haplotype analysis. The results showed that carriers of the T_a_-G_b_ haplotype had a 2-fold increased risk for BA compared with non-carriers. However, the two SNPs were not in LD (*r^2^* = 0.16 in the HapMap database; *r^2^* = 0.173 in our study). Based on the conclusion of the research conducted by Cheng, et al. [16], namely, that the risk allele was associated with a reduced expression level of *ADD3*, we hypothesized that the T allele of rs17095355 may play an integral role in BA susceptibility and *ADD3* transcription. In other words, we hypothesized that the T allele may be associated with decreased *ADD3* transcription, which in turn produces lower levels of the *ADD3* protein in the CT and TT genotypes or in the T_a_-G_b_ haplotype carriers than in the non-carriers; thus, the insufficient *ADD3* protein levels lead to the BA phenotype. However, we did not have a large enough sample size to estimate whether the C allele of the rs10509906-containing haplotype, as a protective allele carried by individuals, might enhance the transcriptional activity of *ADD3* and thus prevent the phenotype that usually results from a low level of *ADD3*. Therefore, the haplotype construction may suggest that the two SNPs together tag a third, untyped, SNP associated to BA, to some extent, probably revealed increased risk in the BA susceptibility. Above all, these results suggested that the *ADD3* gene was associated with the pathogenesis and development of BA.

However, several limitations should be noted in our study. The sample size was relatively small. BA is a complex trait resulting from both environmental and genetic factors. The environmental factors and rare genetic variations associated with BA have not yet been identified, which limited further investigation of the gene-environment interactions. In the absence of functional experiments, it is unclear whether these two SNPs are causally related to BA. Hence, fine-mapping of 10q24.2 region and functional experiments is warranted to identify causal variant.

In conclusion, the results from our study in a Chinese population verified the effective role of rs17095355 in BA susceptibility and suggested that the variant of rs17095355 was also associated with an increased BA risk. The haplotype analysis revealed that the T_a_-G_b_ haplotype in *ADD3* may be a genetic BA susceptibility factor.
